# Sub-millisecond closed-loop feedback stimulation between arbitrary sets of individual neurons

**DOI:** 10.3389/fncir.2012.00121

**Published:** 2013-01-10

**Authors:** Jan Müller, Douglas J. Bakkum, Andreas Hierlemann

**Affiliations:** Bio Engineering Laboratory, ETH ZürichBasel, Switzerland

**Keywords:** closed-loop, high-density microelectrode array, STDP, acausal stimulation, LTD, sub-millisecond

## Abstract

We present a system to artificially correlate the spike timing between sets of arbitrary neurons that were interfaced to a complementary metal–oxide–semiconductor (CMOS) high-density microelectrode array (MEA). The system features a novel reprogrammable and flexible event engine unit to detect arbitrary spatio-temporal patterns of recorded action potentials and is capable of delivering sub-millisecond closed-loop feedback of electrical stimulation upon trigger events in real-time. The relative timing between action potentials of individual neurons as well as the temporal pattern among multiple neurons, or neuronal assemblies, is considered an important factor governing memory and learning in the brain. Artificially changing timings between arbitrary sets of spiking neurons with our system could provide a “knob” to tune information processing in the network.

## Introduction

Different theories describing learning and memory in the brain have been developed, and converging evidence shows that the precise activity timing of individual or groups of neurons may play a paramount role in plasticity of neuronal circuits. The well-known spike timing dependent plasticity (STDP) rule states that if two synaptically connected neurons fire within tens of milliseconds of each other, the connectivity strength of the involved synapses gets potentiated or depressed depending on the firing order. In pioneering studies, STDP rules were discovered (Markram et al., 1997) and further characterized (Bi and Poo, 1998; Song et al., 2000) by observing the effect of correlated firing of two neurons either artificially induced by stimulating a pre-and a post-synaptic neuron with two patch-clamps or by applying trains of paired-pulse stimuli to one neuron in the network (Bi and Poo, 1999). Furthermore, computation in a network is likely due not only to the relative timing of two individual neurons but also to the correlated activity of different neurons forming an associated group, i.e., assembly (Chang et al., 2000; Izhikevich, 2006). In this vein, different studies reported the existence of precise time-locked activity patterns of multiple neurons, both *in vivo* and *in vitro* (Abeles and Gerstein, 1988; Bienenstock, 1995; Ikegaya et al., 2004; Rolston et al., 2007). Having a system to generate feedback stimulation quickly and accurately to interact with such activity patterns would expand such studies beyond finding rules governing the plasticity between two cells toward finding rules governing the spatio-temporal dynamics of whole networks or assemblies (Froemke and Dan, 2002; Izhikevich et al., 2004).

In recent years, different systems to artificially control such feedback stimulation in a closed-loop manner, and thus study neuronal plasticity, have been developed for both *in vivo* (Jackson et al., 2006b; Bontorin et al., 2007; Venkatraman et al., 2009) and *in vitro* applications (Bontorin et al., 2007; Hafizovic et al., 2007; Novellino et al., 2007; Rolston et al., 2010; Zrenner et al., 2010; Wallach et al., 2011). In turn, activity-dependent feedback stimulation was shown to modify the functional connectivity of neuronal networks, both *in vivo* and *in vitro*, as done by reprogramming the motor output of freely behaving primates (Jackson et al., 2006a), changing the functional connectivity in rat forelimb sensorimotor cortex (Rebesco et al., 2010), or shaping *in vitro* neocortical networks into predefined activity states (Bakkum et al., 2008b). *In vivo* systems usually record from needles inserted into a certain location of the brain and subsequently stimulate the same or another site upon the detection of activity. These systems usually comprise the implanted needles, a head stage to amplify the signals, and some means to transmit the acquired signals to a PC. In the case of closed-loop feedback stimulation, these systems usually feature a dedicated very-large-scale-integrated application-specific circuit (VLSI ASIC) (Chen et al., 2009; Rizk et al., 2009; Lee et al., 2010; Azin et al., 2011), or use a general-purpose microcontroller to achieve the respective goals (Mavoori et al., 2005; Zanos et al., 2011). Most *in vitro* systems, on the other hand, use a data acquisition card (DAQ) to sample data for analysis on a PC; feedback stimulation is typically returned through a DAQ card as well.

**Figure 1:**
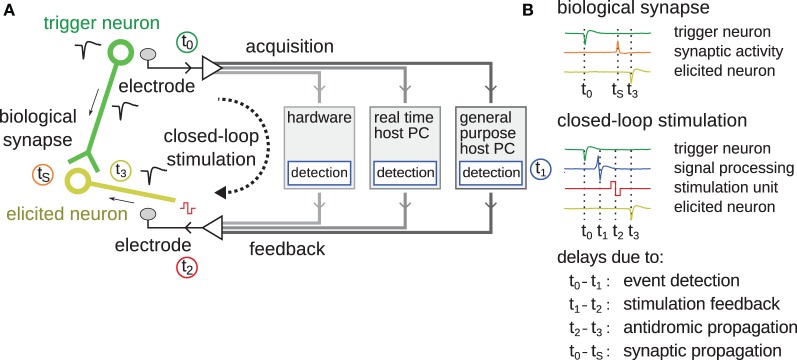
**Schematic overview of latencies in feedback stimulation systems. (A)** The different components making up a closed-loop feedback stimulation system are shown. The green circle represents the “trigger neuron” whose action potential initiates the start of the loop. The green line represents an axon connecting to synapses of the elicited neuron drawn in yellow. The black dashed arrow shows the closed-loop feedback stimulation path. Between data acquisition and stimulation feedback, different components, over which the feedback-loop can be closed, are possible, including digital signal-processing hardware, a real-time host PC, or a general purpose host PC. The time points *t*_0-3_ and *t*_S_ correspond to different events as listed in **(B)**, such as the occurrence of the spike; its detection after signal-processing; the stimulation feedback; and the antidromic propagation of an action potential back into the soma of the elicited neuron. At time *t*_S_, the synapse activates due to pre-synaptic activity of the trigger neuron. The color of the traces corresponds to the color of the timings of *t*_0-3, S_ and schematically shows the timeline of the respective signals.

In order to accurately control the timing of feedback stimulation loops within the timescales relevant for STDP to occur, the delays introduced by a system must be understood. A generic description is given in Figure 1. Different system implementations will have different sources for and values of delays. Signal-processing algorithms introduce an inherent delay in the processing itself. Systems, which rely on general-purpose computers, might introduce latencies and jitter through the presence of data buffers, interrupts, shared resources, or user interactions, etc. In Figure 1, the time points *t*_0−3_ and *t*_S_ specify the occurrence of important events. At *t*_0_ = 0, the trigger neuron emits an action potential, which is recorded by the acquisition system. After entering the signal-processing stages, it is ready to be detected as a spike event at time *t*_1_. From there, the system emits a stimulation pulse hitting the electrode at time *t*_2_. Conventionally, the loop is considered “closed” at this point. The stimulation pulse evokes neuronal activity, frequently activating nearby axons (Bakkum et al., 2008a) whose signals propagate antidromically toward the soma until eliciting an action potential at time *t*_3_. In the case depicted in Figure 1, where the trigger neuron is synaptically connected to the elicited neuron, an additional biological time, *t*_S_, denotes the duration of an action potential propagation through the axon of the trigger neuron until synaptic activation of the elicited neuron. In case where *t*_0_−*t*_1_−*t*_2_ is faster than *t*_0_−*t*_S_, that is when the signal propagates faster through the artificial feedback-loop than down the axon toward the biological synapse, acausal stimulation, and thus the introduction of long-term depression (LTD) according to the STDP rule, is possible.

In order to apply closed-loop stimulation feedback precise and fast enough to study plasticity at the timescales of STDP or acausal stimulation, and flexible enough to interact with cell assemblies, we developed a field-programmable gate array (FPGA)-based system, interfaced with a complementary metal–oxide–semiconductor high-density microelectrode array (CMOS-MEA). The CMOS-MEA features a total of 126 readout and 42 stimulation channels, which can be connected to an almost arbitrary subset of 11,011 5 × 7 μm^2^ electrodes, arranged in a 2 × 1.75 mm^2^ array. The feedback stimulation loop is closed around the CMOS-MEA using an FPGA that performs signal-processing, such as spike-detection and feedback generation. The system functionality was verified using cultured networks of cortical neurons and glia. The minimum programmable latency of the closed-loop stimulation feedback (*t*_0_−*t*_1_−*t*_2_) was 400 μs with jitter below 50 μs, suitable to induce STDP. This is faster than many axonal propagation delays (*t*_0_−*t*_S_), rendering it possible to conduct acausal stimulation experiments. An “event engine” was designed and implemented to trigger feedback stimulation at the occurrence of activity patterns, such as those described in Ikegaya et al. (2004) and Rolston et al. (2007). Patterns could be of almost arbitrary length and could consist of up to 1000's of individual elements, only limited by the available resources of the FPGA. Configurations for the event engine could be (re)loaded within milliseconds. Unique to this system is the possibility to enable low-latency, high-throughput, STDP-like experiments as well as acausal stimulations across many individual neurons, or neuronal assemblies in parallel through the simultaneous application of many feedback stimulation loops. To infer changes in synaptic strengths, correlations between putative mono-synaptically connected neurons (Fujisawa et al., 2008) can be monitored using extracellular spikes. In the future, high-throughput STDP experiments will be possible by adding a patch electrode to the system in order to monitor changes in intracellular post-synaptic currents.

## Methods

### System architecture

The main design goals were to implement (1) multiple feedback stimulation loops (2) to match arbitrary spike patterns with (3) short latencies (<1 ms) and (4) high accuracy (<50 μs) (5) while still recording from all available 126 channels. A main component of the presented system is an FPGA, used to hijack signals traveling between the analog-to-digital converter on the CMOS device and the host PC. Due to the inherent parallel nature of FPGAs, signal-processing and feedback generation using data from additional recording channels can be done without introducing additional delays or jitter.

**Figure 2:**
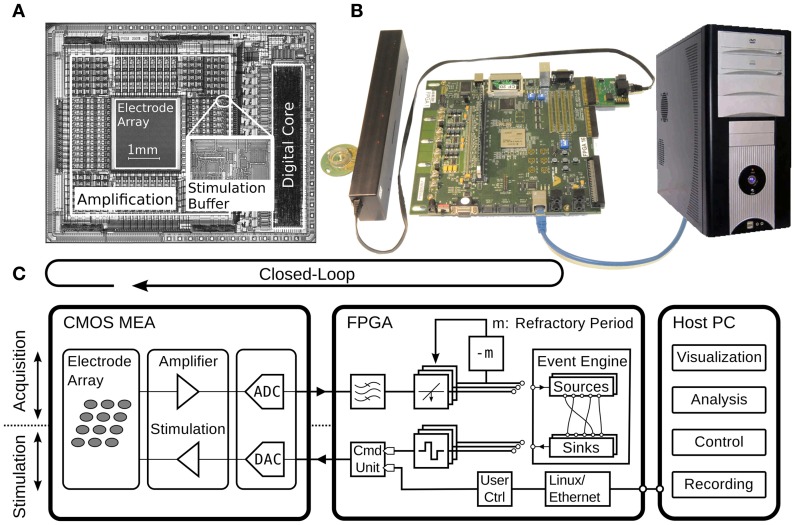
**Overview of the presented closed-loop system, implemented with a CMOS-MEA, an FPGA, and a host PC. (A)** Micrograph of the CMOS-MEA highlighting the electrode array, amplification and stimulation units, and the digital core with an inset showing a close-up of the stimulation buffer. **(B)** Photograph of the CMOS-MEA plugged into the custom printed-circuit board, which is connected through an LVDS link to the Xilinx Virtex II pro FPGA board from Digilent Inc., Pullman, USA. The host PC running data acquisition and visualization software is connected to the FPGA through Ethernet. **(C)** Schematic diagram of the setup. The diagram shows the acquisition (upper part) and stimulation path (lower part). The feedback stimulation loop is closed around the CMOS-MEA and the FPGA. The components are described in detail in the text.

The system consists of three main parts as shown in Figure 2. The first is a high-density CMOS-MEA device featuring on chip signal-conditioning, stimulation, and analog-to-digital conversion (ADC) units (Frey et al., 2010), described in more detail in the next section. It is plugged into a custom printed circuit board (PCB) that provides reference voltages and clock signals. The digital data as provided by the CMOS-MEA are transmitted through a low-voltage differential link to reduce sensitivity to electromagnetic interferences as caused, for example, by a nearby incubator. The second part is an FPGA, which reads in the differential signals and subsequently performs signal-processing, spike-detection, and feedback stimulation, as well as compression and framing of the data to be sent via TCP/IP over Ethernet to a host PC, the third main part. On the host PC, further data analysis can be performed online or offline. It is also used to program and control the CMOS-MEA device during experimentation with different settings, like amplifier gain or electrode-to-amplifier routing, in order to be adopted for use in different experimental sessions.

### CMOS device

The CMOS-MEA includes 126 readout channels with programmable amplification (0 dB to 80 dB), on chip ADCs sampling at 20 kHz, and stimulation capabilities (see below). It features a sensor area of 2 × 1.75 mm^2^ with a total of 11,011 electrodes, each with a size of 5 × 7 μm^2^ and a pitch of 18 μm. Beneath the electrodes resides a sophisticated analog-switching matrix to connect an almost arbitrary subset of the 11,011 electrodes to the 126 readout channels. The readout electronics were placed outside of the sensor array, instead of directly below the electrodes as done in active-pixel sensor devices (APS) (Berdondini et al., 2009), to provide space for larger circuitry elements that produce less noise. This scheme also allows for reducing the pitch of the electrodes below the spatial requirements of the readout electronics. See Frey et al. (2010) for more details.

### FPGA

A reprogrammable Virtex II pro FPGA (Xilinx Inc., San Jose, USA) was used as an intermediate signal-processing device between the CMOS-MEA and the host PC to perform real-time signal-processing, decision-making and feedback generation. The FPGA acquires digital data coming from the differential link and forwards it to a PC over Ethernet. The Virtex II pro features an embedded PowerPC microprocessor running at 300 MHz that operates a Linux kernel with a Busybox operating system. The TCP/IP stack of the Linux kernel handles the network communication and data transfer. As the embedded PowerPC microprocessor is relatively slow, compared to modern CPUs, this provides a bottleneck for fast data transmission. We measured the latency between the TCP/IP stack of the FPGA and the host PC to be 83 ± 21 ms (mean ± SD, *N* = 308) at full-frame data transmission, which is larger than the STDP window of up to tens of milliseconds. One solution to this problem might be to stop streaming of the full data readout, while performing a closed-loop experiment and to only route out the data channels strictly needed for the closed-loop feedback stimulation. This would free some of the bandwidth of the Ethernet link and make it available for faster feedback stimulation. Crucially, however, we would lose the possibility to simultaneously monitor neural activity elsewhere in the cultured network by applying such a paradigm. Another option might be to bypass the Ethernet link by streaming the data directly to a DAQ card, attached to the host PC, and to send stimulation information back through a second link to the FPGA. All these methods are less practical than using the universal TCP/IP connection, which plugs into almost every kind of host PC and does not require additional hardware. An attractive alternative for achieving low latencies was to implement all needed signal-processing and feedback generation directly on the FPGA. The next paragraphs highlight the different building blocks needed to implement such a scheme. Although the FPGA can be reprogrammed at will, this is time-consuming and error prone and, therefore, not suitable during an experimental session. To accommodate reprogramming, a more flexible, module-based design was developed in VHDL and programmed into the FPGA logic together with a software interface to quickly reconfigure the connectivity of the individual modules (see “Event Engine”).

### Spike-detection

One such signal-processing building block is spike-detection, which extracts spiking events from the raw voltage traces, recorded at the electrodes. Spike-detection is implemented as a threshold crossing. The signals are first digitally band-pass filtered with a two-tab Butterworth filter (500 Hz–3 kHz) to suppress DC offset components and higher frequency noise; this will emphasize the action potential frequency components. The detection threshold level is user-programmable and typically set around 4.5 times the noise standard deviation. During experimentation, this value can be determined by software running online on the host PC. After an identified spike event, we set a programmable refractory period to 3 ms. After stimulation, detection was disabled for 3 ms as well, to avoid oscillating loops due to feedback stimulation artifacts being falsely classified as spikes.

### Event engine

**Figure 3:**
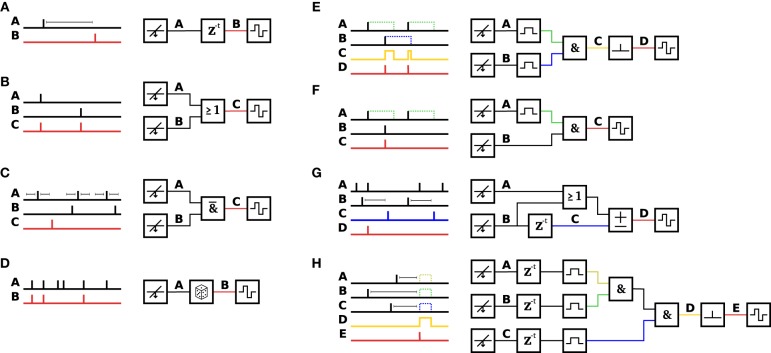
**Example configurations of the event engine.** Stitching together the appropriate set of modules allows the event engine to be configured to match a variety of patterns in order to trigger feedback stimulation. Different minimal examples are shown. **(A)** A DELAY element is inserted after a DETECTION module to trigger STIMULATION after a programmable delay. This configuration, with the delay set to zero, was used for the experiments shown in Figures 5, 7. **(B)** Either an event on channel **A** OR an event on channel **B** triggers stimulation. **(C)** In a programmable time window before and after an event on channel **A**, there may not be any event on channel **B** in order to trigger stimulation (trace **C**). **(D)** A RAND module propagates or discards the events, in this case with a probability of ½. **(E)** Events on channel **A** and channel **B** are fed through SPREAD modules into an AND module, which outputs events (on trace **C**), when both inputs are active. The intermediate trace **C** is fed into a SPREAD^-1^ module to trigger stimulation at the onset of the event. **(F)** When the event on channel **B** happens subsequently to an event on channel **A**, an event **C** is generated **(G)** An ACCU module is set to increment, when either an event on channel **A** OR channel **B** happened, and to decrement, when a delayed event from channel **B** (trace **C**) arrived. In this example, the ACCU threshold is set to three events. Once the threshold is reached, the internal counter gets reset to zero. When the three input events happen shortly after each other, a stimulation event gets emitted. As shown in the example, the delayed channel **B** (trace **C**) decrements the accumulator and thus delays or prohibits crossing of the threshold. **(H)** All modules can be combined together to achieve almost arbitrarily complex pattern matching. For example, this configuration was used to match the pattern of Figure 6. The formula describing this pattern is: STIMULATION(*1*, SPREAD^-1^(AND(AND(SPREAD(*2 ms*, **A**), SPREAD(*2 ms*, **B**)), SPREAD(*2 ms*, **C**)))).

To avoid time-consuming reprogramming of the FPGA fabric, a more flexible and modular event-based scheme for feedback generation (Event Engine) was designed and implemented. The event engine consists of small building blocks, called modules, each of which implements a specific simple function. Each module has one or more event sinks as inputs and one event source as an output. By connecting the event sources to the appropriate event sinks, different, almost arbitrary pattern matching, and event handling algorithms can be achieved. Table 1 summarizes the implemented modules. Figure 3 shows different basic configurations to achieve defined pattern matching. In Figure 3A, the simplest closed-loop configuration is depicted, where the source of a spike-detection module gets connected to the sink of a delay unit and from there to a stimulation function generator. Whenever the source produces an event (i.e., in this case detects a spike), the sink triggers a stimulation pulse after a defined time delay. By means of software, the sources can be connected to sinks dynamically and rapidly within milliseconds while running an experiment such that pattern matching can adapt to ongoing activity in the living culture. One notable property is the lack of time binning. Each spike gets represented as a single pulse with a temporal resolution set by the sampling frequency, i.e., 20 kHz. As a consequence, certain desired operations might not make sense, as the biological neurons have some inherent variability in when they spike. For example, the user might want to match a pattern, where two neurons spike together (see Figure 3E). To achieve this, a SPREADING module “spreads” the spike pulse in time in order to compensate for jitter. This way, the subsequent AND module can generate an output event whenever the two neurons fire together within a specified range of time. As discussed in Ikegaya et al. (2004) and Rolston et al. (2007), 2 ms is suitable for most recurring patterns. Another module can be used to convert the spread-out spike pulse back into a single one-shot event, which then can be used, for example, to trigger the stimulation unit only once per spread-out pulse. The particular selection of implemented modules (as listed in Table 1) represents a minimal set, which, if combined in the appropriate way, allows for matching different kinds of events, such as specific spatio-temporal activity patterns, time sequences, network bursts, local bursts, etc. In order to keep the event engine as flexible as possible and adaptable to different, possibly unforeseen pattern matching sequences, the implementation of a minimal set of small building blocks has been chosen over the approach, where each envisioned pattern would require a single, but more complex, and less flexible building block. Thus, available modules can be combined together in almost infinite different ways, limited only by the available FPGA memory that keeps track of all source-sink associations.

**Table 1:** **A minimal set of modules making up the event engine**.

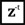	DELAY(*t*, **A**)	Delays the event **A** by a defined amount of time *t*.
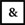	AND(**A**, **B**)	Emits an event, when both of the two input events, **A** and **B** occurred simultaneously.
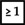	OR(**A**, **B**)	Emits an event, when either of the two input events **A** or **B** occurred.
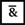	INH(*t*, **A**, **B**)	Emits an event, when an event on **A**, however, no event on **B** occurred in a defined time window, *t*, in order to create inhibitory feedback-loops.
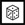	RAND(*p*, **A**)	Propagates the event **A** to the output or drops it after a Bernoulli-distributed pseudo-random variable with a definable probability, *p*.
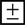	ACCU(*n*, **A**, **B**)	Increments (event **A**) or decrements (event **B**) an internal accumulator and emits an event after a definable threshold, *n*, has been reached, after which it is reset to zero.
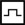	SPREAD(*t*, **A**)	Spreads the event **A** in time for a defined time, *t*.
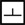	SPREAD^–1^(**A**)	Converts the onset of a spread-out event **A** back into a single event.
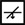	DETECTION(*c*)	Emits an event, when the specified channel, *c*, detected a spike.
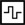	STIMULATION(*c*, **A**)	Generates a stimulation pulse on the specified channel, *c*, whenever input event **A** happened.
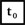	START	Single pulse after system start-up, which can be used to start repetitive stimulation protocols.

Configurable parameters are represented in italics (t, p, n, c), and input events are denoted in bold letters (**A**, **B**).

### Stimulation/function generator

The CMOS-MEA has 42 on-chip integrated stimulation units, which are driven by two 10bit DACs. On the FPGA is a function generator implemented to achieve arbitrary stimulation waveforms. A defined waveform has to be programmed at the start of the experiment. We used biphasic, first positive then negative voltage pulses of 200 μs duration per phase and ±300 or 400 mV amplitude. The stimulation buffers can be chosen to operate in voltage- or current mode (Livi et al., 2010). Whenever the event engine outputs an event, the appropriate stimulation buffer, located on the CMOS-MEA, gets connected, and the function generator starts its operation. Stimulation artifacts on the readout channels could result in falsely detected spikes and cause a reverberation problem for low-latency feedback-loops. Therefore, spike-detection is blanked during a time period of a few milliseconds after stimulation onset.

### Cultures

The performance of the closed-loop system was tested with cortical neurons and glia grown over the CMOS-MEA. Animal handling protocols were approved by the Basel-Stadt Veterinary office according to Swiss federal laws on animal welfare. Briefly, a time-pregnant rat was anesthetized using isoflurane, then decapitated to gain E18 embryos. Cortices were extracted from the embryos and dissociated enzymatically in trypsin (Invitrogen) followed by mechanical trituration. A layer of laminin (Sigma) over a layer of poly(ethyleneimine) (Sigma) was used to adhere between 20 and 40 k cells. Plating media consisted of 850 μL of Neurobasal, supplemented with 10% horse serum (HyClone), 0.5 mM GlutaMAX (Invitrogen), and 2% B27 (Invitrogen). After 24 h, the plating media was changed to growth media: 850 μL of DMEM (Invitrogen), supplemented with 10% horse serum, 0.5 mM GlutaMAX, and 1 mM sodium pyruvate (Invitrogen). Cultures matured for 3–4 weeks prior to experimentation, and experiments were conducted inside an incubator to control environmental conditions (34.5°C and 5% CO_2_). For further details see Hales et al. (2010).

## Evaluation and results

This section begins with data characterizing the suitability of our setup to perform closed-loop feedback stimulation experiments, using cultures of cortical neurons and glia for validation. First, the process of identifying neurons to be used in closed-loop feedback stimulation will be described. Then the system's loop speed and jitter performance will be quantified. An example event engine was run to provide stimulation feedback, triggered by an activity pattern. Preliminary data and techniques to analyze the consequences of such stimulation on the functional connectivity between neurons will be presented and discussed. Finally, an experimental session to induce LTD through acausal stimulation will be sketched, and its implications discussed. Data in the figures demonstrate proof-of-principle experiments from individual cultures, the setup has, however, been successfully applied to many tens of cultures.

### Recording/stimulation selectivity

**Figure 4:**
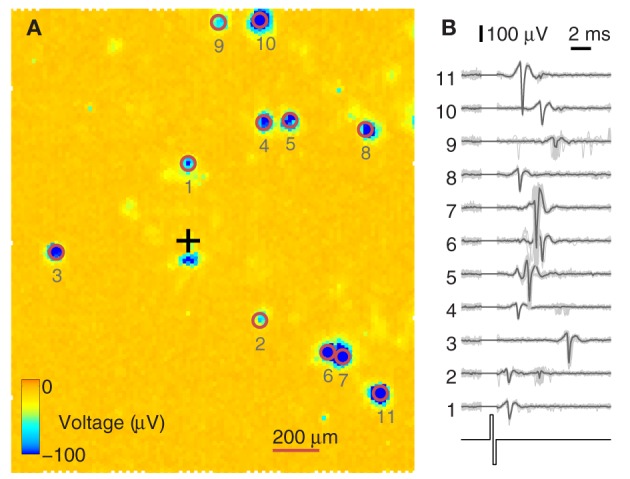
**Identification of directly evocable action potentials. (A)** Data recorded in response to repeated stimulation of one electrode (black cross) from the whole 2 × 1.75 mm^2^ sensor area of the CMOS-MEA (each pixel is one electrode). Recording electrode configurations were scanned across the array in sets of 126 electrodes at a time. For every configuration, data were recorded for 12 ms after stimulation onset. The amplitude of the negative voltage peak within these 12 ms is color-coded and clipped at –100 μV. Blue indicates the detection of directly evoked somatic action potentials. **(B)** Example traces from 11 somas and the stimulation pulse are shown on the right. Traces from 30 stimulation trials are overlaid, with the median trace highlighted in black. The stimulation artifact was blanked prior to recording. Numbers are ordered by increasing distance from the stimulation site.

High-density CMOS-MEAs can potentially sample from complete neuronal populations. Due to the high-density (18 μm pitch) of the CMOS electrode array, every neuron lying on the 2 × 1.75 mm^2^ array can be bidirectionally addressed. On the other hand, when stimulating one electrode, a defined subset of neurons is often directly activated in response (Bakkum et al., 2008a). Figure 4 shows such a scenario. In Figure 4A, one electrode, marked with a black cross, was stimulated multiple times, and the evoked activity was recorded during a window of 12 ms after stimulation onset. The median calculated over all voltage traces filters out noise and spontaneously spiking neurons/traces. Reliable activity (usually with a jitter on the order of 100 μs or below) is considered due to an antidromic action potential initiated at the neuron's axon (Lipski, 1981). Since only a subset of 126 out of the 11,011 electrodes can be readout simultaneously, the stimulation sequence was repeated multiple times, each time with a different subset of electrodes, until all electrodes were covered. After recording all sequences, the traces of the individual recordings were aligned in time. To highlight the electrodes that recorded elicited action potentials, the negative peak of the recorded voltage level during 12 ms after stimulation is color-coded and clipped at −100 μV. The red circles around the exemplified 11 spots highlight neurons that fired directly elicited action potentials. Their traces are individually shown in Figure 4B, demonstrating that the elicited action potentials were reliably and precisely fired after a given time, and only in a few cases (traces 2, 4, 6, 9), activity with different timing occurred. These could stem from a different neuron that happened to sit near the same electrode and/or from action potentials occurring within a coincident network burst.

As shown, recording and stimulation with the CMOS-MEA feature high spatial resolution and, therefore, are locally very confined. However, the facts that one electrode can detect signals from more than one neuron, and that the stimulation through one electrode can directly evoke action potentials of more than one neuron have to be considered when planning closed-loop feedback stimulation experiments. In this case, the feedback-loop is not closed between two neurons, but includes two sets of neurons.

### Feedback latencies

According to the rules of STDP, the timing window to induce long-term potentiation (LTP) at synapses is between less than a few milliseconds and up to tens of milliseconds post-synaptic activation before and after pre-synaptic activity. Thus, even though feedback cycles of 5–10 ms are fast enough to induce LTP, we aimed at reaching cycle-times below 1 ms to enable the system to perform acausal stimulations, as explained in the respective section below.

**Figure 5:**
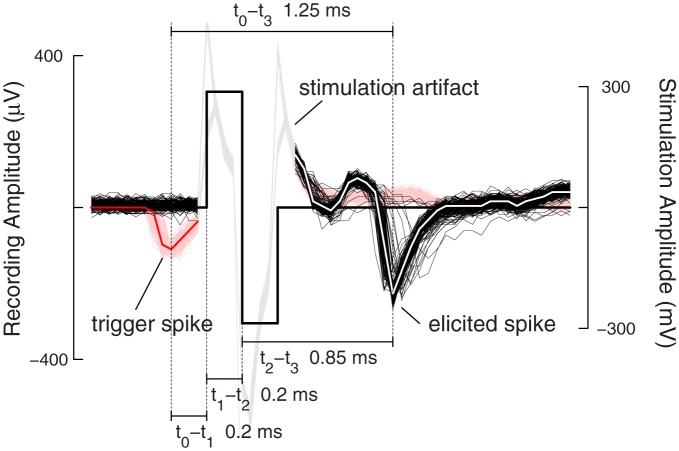
**Feedback stimulation performance.** One hundred and twenty-eight traces from a closed-loop stimulation sequence are aligned at the stimulation onset-time and overlaid. Traces in red show the trigger spikes with the median over all trigger traces shown in bold red. The stimulation artifact is grayed-out for better visual clarity. The traces in black show spikes, elicited in all but four cases after stimulation. The median over all elicited traces is shown in bold white. The antidromic propagation delay for the elicited spikes was around 0.85 ms. The different timings, detection delay, stimulation delay, and antidromic propagation delay sum up to the full loop delay of 1.25 ms.

Figure 5 shows the overlay of 128 traces of the feedback-loop. Here, the event engine was configured to detect events on only one channel and stimulate immediately after detection, i.e., without any further delays in order to test the system performance (cf. Figure 3A). The traces are aligned at the onset-time of the stimulation pulse, and time zero is set to be at the negative peak of the spike of the trigger neuron. In red are the traces from the trigger neuron, and in black, the traces from the elicited neuron. The timing between a trigger neuron spike and the onset of the stimulation pulse was 200 μs, i.e., 4 sampling periods. This delay arises as follows: 50 μs (1 sampling period) was used to buffer the incoming data in the FPGA; 100 μs accounted for the delay of the two-tab Butterworth filter and the last 50 μs account for all other delays, such as synchronizing the stimulation pulse with the recording sampling time. Delays for sending digital data between the CMOS device and the FPGA were on the order of nanoseconds and thus are negligible. When stimulating with biphasic voltage pulses, the steep negative transition, which injects negative current (I = C × dV/dt), is the time point, when a cell is activated (Wagenaar et al., 2004; Bakkum et al., 2008b). Thus, this time point was taken to measure the latency between stimulation and an elicited spike. In the case depicted in Figure 5, this timing is 0.85 ms, and the overall latency between trigger neuron activity and a spike on the elicited neuron was 1.25 ms.

As can be seen in Figure 5, besides achieving short feedback cycles, another advantage of using digital hardware (in this case FPGAs) for feedback generation is that no additional jitter is introduced, as such a system is fully deterministic. Sources of jitter in other systems (Hafizovic et al., 2007; Rolston et al., 2010) that close the feedback-loop around general-purpose or real-time personal computers are, for example, system interrupts that might disrupt the data processing, or buffer sizes of the USB, TCP/IP, or DAQ cards, which have to be set large enough in order to guarantee full data throughput. Usually these buffers have a size larger than one sample period. Depending on when an event happened inside this buffer, the latency could be larger or smaller and thus introduce jitter. This can be avoided by using digital hardware to hijack the data stream. In our case, the jitter was below ±50 μs and arose from the fact that neural activity is, of course, not aligned to the sampling period of the CMOS-MEA (50 μs). The exact time of the threshold crossing relative to the negative spike peak depends, among other things, on the slope of the spike waveform. Since the recorded signal was not interpolated between samples, this was an unavoidable source for jitter.

### Pattern matching

**Figure 6:**
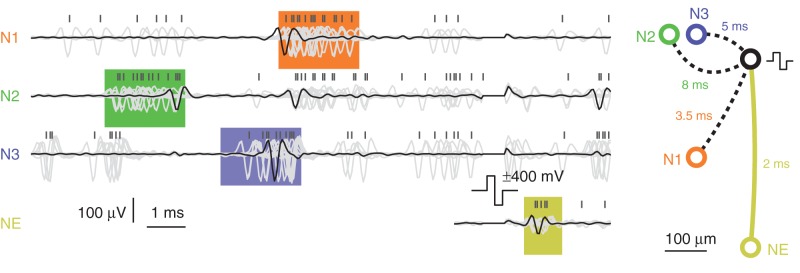
**Pattern-matching feedback stimulation.** Electrode traces were recorded from neurons sitting on three different electrodes N1–N3 while performing pattern matching. The pattern was matched 22 times within 12 min, all overlaid and drawn in light-gray color. One arbitrary pattern is highlighted with black traces. The 12 ms before and 4 ms after stimulation pulse are shown. The orange, green, and blue colored boxes represent the spread-out-windows set in the event engine. A yellow box of arbitrary width is drawn around the elicited activity of neuron NE. Above the traces, negative peak times are marked with black vertical bars, showing spikes clustered within the colored boxes. The figure on the right shows electrode locations and the timings making up the pattern to match as well as the antidromic propagation delay of 2 ms to the elicited neuron.

To demonstrate the event engine in operation, feedback stimulation, triggered by an activity pattern, was performed. For the dataset presented in Figure 6, the event engine was programmed according to Figure 3H and classified spontaneous activity patterns as follows: A neuron recorded on electrode N2 fires an action potential; then an action potential is recorded from a neuron on electrode N3 after 3 ms; finally an action potential is recorded on electrode N1 after another 1.5 ms. Each individual event occurrence was allowed to have a jitter of ±1 ms. After successful identification of such a pattern, a stimulation pulse was emitted to elicit action potentials on a different neuron, NE. The cell cultures under investigations typically expressed bursting behavior, and this was when almost all of the patterns occurred. During bursts, the cells usually fired more than once at an elevated frequency, and this explains why the neurons on electrodes N1–N3 showed additional spikes “outside” of the detected pattern. Nevertheless, the pattern matching event engine identified 22 activity pattern occurrences during 12 min of recording.

### Correlation analysis

**Figure 7:**
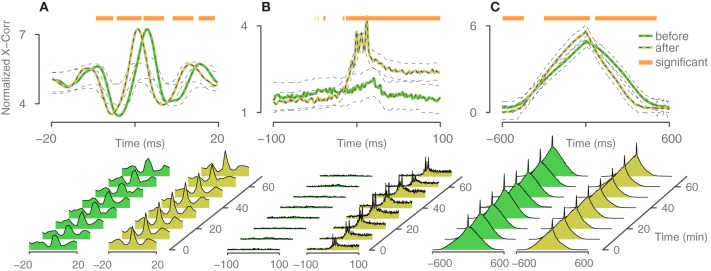
**Cross-correlation analysis.** Three descriptive cases of changes in correlated firing between trigger neurons and elicited neurons. Spontaneous activity was recorded 1 h before and 1 h after the application of closed-loop feedback stimulation. Periods, where the difference exceeded a confidence bound (see text), were assigned to be significant and are indicated with an orange bar. The 95% confidence intervals are indicated with black dashed lines. Cross-correlation is computed based on trains with 9000–13000 spikes per neuron. **(A)** Relative probability remained constant, but the timing between trigger neuron and elicited neuron changed and became more synchronous. **(B)** The elicited neuron became more likely to fire in concert with the trigger neuron. **(C)** Relative timing within a network burst changed.

To assess the connectivity between different neurons and the efficacy of change, induced by the closed-loop feedback stimulation, cross-correlation curves (Perkel et al., 1967) were computed between spike trains of the trigger neuron and the elicited neurons. When exceeding a 95% confidence interval (Brillinger, 1976), correlation is considered significant. Figure 7 shows three descriptive cases, comparing the cross-correlation curves from 1 h of spontaneous activity before and after closed-loop feedback stimulation was applied for 1 h. To evaluate significance of the change, a similar procedure as in Fujisawa et al. (2008) was used. Briefly, the two times 1 h of spontaneous activity recordings were divided into smaller bins of 10 min duration and were randomly assigned to be before or after the closed-loop stimulation. Cross-correlation from this shuffled data was computed for both “before” and “after” and the difference was evaluated. This procedure was repeated 1000 times to generate a surrogate data set. Points on the *x*-axis, where the true difference is larger than 95% of the surrogate data, were assigned to be significant and are marked with an orange bar in Figure 7. Assessing the true connectivity of neuronal networks by means of extracellular measurements is difficult, and using the cross-correlation to that end is not ideal, as effects like common inputs or firing rate changes cannot be easily explained. However, in our context of evaluating the effect of feedback stimulation, we do not necessarily seek to precisely explain the changes in network connectivity, but to rather demonstrate that a change occurred at all and to what extent.

### Acausal stimulation

**Figure 8:**
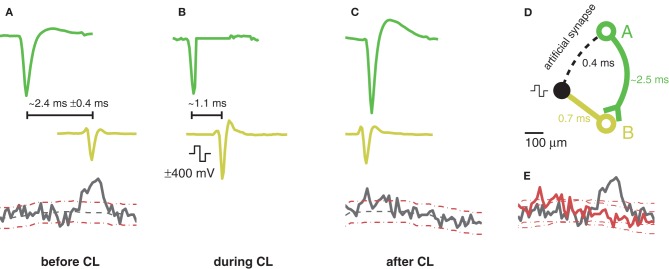
**Schematic of an acausal stimulation sequence. (A)** Spontaneous activity before application of the closed-loop. Shown spike traces are the median waveform of several spikes aligned at the negative peak. Top: Spike trace of the trigger neuron, A, in green. Middle: Example spike trace of a correlated neuron, B, drawn in yellow. The time delay between the plotted spikes of neuron A and neuron B was chosen to align with the maximum peak of the cross-correlation curve. Bottom: Cross-correlation curve of spike-times of neuron B with respect to neuron A. 95% confidence intervals are drawn with dotted red lines. Cross-correlations were computed with trains having 2000–3000 spikes. Significantly elevated correlated activity of neuron B can be detected around 2.4 ± 0.4 ms after neuron A fired an action potential. **(B)** Same situation as in **(A)** but with a closed-loop feedback stimulation applied. Due to the low-latency loop, the time delay of the yellow spikes with respect to the green ones was reduced by about 1.3 ms. For neuron A, the trace was zeroed at the start of the stimulation pulse. **(C)** Same as **(A)** but after the application of the closed-loop feedback stimulation. The cross-correlation no longer shows a significant peak for latencies larger than zero. The time delay between the plotted spikes of neuron A and neuron B was again chosen to align with the maximum peak of the cross-correlation. **(D)** Geometric sketch of the situation. The trigger neuron A and its axon are shown in green and the elicited neuron B in yellow. **(E)** Comparison of the two cross-correlation curves before (black) and after (red) the acausal stimulation with their 95% confidence intervals.

One motivation for very short feedback cycles is to open the possibility of acausal stimulation. If the closed-loop stimulation (*t*_0_−*t*_2_) is faster than the time it takes the action potential to travel along the axon and hit the synapses (*t*_0_−*t*_S_), acausal stimulation and, therefore, induction of LTD by means of closed-loop feedback stimulation is possible. The time that it takes for an action potential, initiated at the axonal hillock, to propagate down the axonal arbor to the synapses depends on the propagation velocity of action potentials along axons and the length of the axons. Action potential conduction velocities in unmyelinated axons were reported around 0.2–0.4 ms^−1^ (Debanne et al., 2011). As demonstrated in Figure 5, the closed-loop stimulation (*t*_0_−*t*_2_) can be as fast as 0.4 ms, meaning acausal stimulation is possible for trigger neurons (*t*_0_) with unmyelinated axons that synapse to an elicited neuron (*t*_3/S_) after a minimum axial length of 80–160 μm. Figure 8 shows such an acausal stimulation procedure. First, before applying a closed-loop, the activity between different neurons was measured then evaluated by computing the cross-correlation. In the example in Figure 8, the firing activity of the second neuron B with respect to the first neuron A was elevated around a delay of 2.5 ms, implying neuron A has a functional connection with neuron B. Integrating the cross-correlation curve, where it exceeds the confidence intervals around 2–3 ms after the reference time zero, reveals an integral probability of around 40% chance for neuron B to spike 2–3 ms after neuron A had fired. Once two such neurons could be identified, closed-loop stimulation can be applied between them with a very short feedback cycle. In the presented example, the delay from the trigger neuron to the elicited spike was around 1 ms, smaller than the average delay between the occurrence of their spontaneous action potentials. The closed-loop feedback stimulation was applied for 20 min, and, afterwards, the correlation was measured again. Now, the correlation no longer exceeded the confidence intervals at around 2–3 ms after the trigger neuron. Note, however, that Bi and Poo (1998) have shown that LTD can only be induced, if the spontaneous synaptic efficiency is not strong enough to evoke a post-synaptic action potential. Otherwise, the post-synaptic Ca^2+^ influx dominates, and LTP will occur. For the experiment shown in Figure 8, the elicited neuron spiked only a fraction of the time, and provided an intermediary synapse; in all other cases, evoked excitatory post-synaptic currents (EPSCs) remained below the threshold. Further experiments are required before drawing conclusions. Additionally, to explore LTD and LTP in more depth, and advantageously, across many synapses simultaneously, extracellular recordings targeted to many trigger neurons, and an elicited neuron on the CMOS-MEA could be combined with an intracellular patch-clamp, attached to the elicited neuron and measuring the incoming EPSCs.

## Discussion

With the presented system, capable of applying multiple flexible feedback-loops simultaneously, many different experiments will be possible. The dynamic clamp technique proved to be a valuable tool for investigating the membrane dynamics involved in action potential generation (Destexhe and Bal, 2009; Economo et al., 2010). In such systems, intracellularly applied closed-loop-controlled voltage feedback enables the manipulation of cell membrane functions. Similarly, extracellularly applied closed-loop stimulation feedback, as presented in this work, might provide a useful tool for investigating cellular and network level plasticity and enable the manipulation of neuronal network functions. Potential questions include how information processing and the amount of memory that can be stored in a cultured network are influenced by adding one or more feedback-loops. Further experiments might involve more detailed studies of both LTP and LTD of individual sets of neurons by implementing causal and acausal feedback-loops between them. Using the pattern matching capabilities of the event engine will allow for extending plasticity studies to the network level. For example, investigations of the temporal order and history of spike trains, similar to those reported by Froemke and Dan (2002) and Ikegaya et al. (2004), could be performed, however, in parallel on multiple different neurons and pathways and, in addition, the respective pathways could be dynamically altered by targeted closed-loop feedback stimulations. Further rules governing plasticity beyond the classical STDP could be investigated.

An inherent limitation of extracellular recording systems is the inability to directly measure EPSCs. Conventional plasticity studies rely on patch-clamp to directly measure the EPSC to assess synaptic connectivity strength. Since these currents are not accessible with extracellular measurement techniques, indirect methods to assess synaptic connectivity have to be employed. Although cross-correlation seems attractive and is commonly used to assess connectivity, either between different brain regions or networks, or even between individual cells, it remains to be investigated to what extent correlation analysis unveils the direct synaptic strength between neurons. A combination of patch-clamp techniques and MEAs would provide a more direct way to measure the EPSC than through the computation of cross-correlation curves. By patching the post-synaptic neuron, EPSC strengths can be directly measured and related to extracellularly recorded pre-synaptic activity. Combining the advantages of both techniques, i.e., the precise EPSC measurements through patch-clamp, and the large-scale parallel, extracellular measurements and stimulations through CMOS-MEAs with flexible feedback-loops programmed by the event engine, would greatly expand experimental horizons. One could study the plasticity of hundreds of synapses in parallel. Furthermore, by hooking up the patch-clamp system to the event engine through dedicated spike-detection and stimulation modules, feedback-loops could be applied through the patch-clamp between extracellularly recorded and intracellularly stimulated (or vice versa) neurons.

Although, due to the high-density of electrodes, potentially all neurons can be read out individually, the recorded signals from two different neurons, located close to each other, are sometimes difficult to separate. A spike-sorting step, incorporated prior to event detection, can help to sort, and separate even neurons recorded from with the same electrodes. This holds in particular for using high-density electrode arrays (Franke et al., 2012). The spike-sorting might enable the identification of neurons with smaller spiking amplitudes, close to the noise level, and the identification of more neurons or cell assemblies. However, a drawback of more sophisticated spike-sorting algorithms is an additional time delay in the detection phase (*t*_0_−*t*_1_). Spike-sorting, together with intracellular stimulation through patch-clamp as described above, could eliminate the aforementioned limitations in section “Recording/stimulation selectivity”: Trigger spikes can be assigned to an individual neuron through spike-sorting, and stimulation pulses will only activate action potentials in the patched neuron.

## Conclusion

By using an FPGA to perform signal-processing, as well as feedback generation, fast, and flexible loop cycles have been realized. Our approach using reconfigurable digital hardware to perform computationally intensive tasks, such as signal filtering, spike identification, decision-making, and feedback generation, is a compromise between traditionally employed methods either using a general-purpose (micro-) processor, which introduces additional latencies, and jitter, and the highly integrated application-specific circuits (VLSI ASICs), which are much less flexible in terms of adaptations to new experimental paradigms. Our achieved closed-loop feedback latencies are lower than many axonal propagation delays and thus enable acausal stimulation. Due to the flexible event engine, high-throughput experiments applying many feedback-loops in parallel are conceivable.

### Conflict of interest statement

The authors declare that the research was conducted in the absence of any commercial or financial relationships that could be construed as a potential conflict of interest.
